# Neurocircuitry of acupuncture effect on cognitive improvement in patients with mild cognitive impairment using magnetic resonance imaging: a study protocol for a randomized controlled trial

**DOI:** 10.1186/s13063-019-3446-9

**Published:** 2019-05-30

**Authors:** Hyo-Weon Suh, Jieun Kim, Ojin Kwon, Seung-Hun Cho, Jong Woo Kim, Hui-Yong Kwak, Yunna Kim, Kyung Mi Lee, Sun-Yong Chung, Jun-Hwan Lee

**Affiliations:** 1 Department of Clinical Korean Medicine, Graduate School, Kyung Hee University, Seoul, 02447 Republic of Korea; 2 Clinical Medicine Division, Korea Institute of Oriental Medicine, Daejeon, 34054 Republic of Korea; 3 Department of Neuropsychiatry, Kyung Hee University Medical Center Korean Medicine Hospital, Seoul, 02447 Republic of Korea; 4 Department of Neuropsychiatry, Kyung Hee University Korean Medicine Hospital at Gangdong, Seoul, 05278 Republic of Korea; 5 Department of Radiology, Kyung Hee University Hospital, Seoul, 02447 Republic of Korea; 6 Department of Radiology, Kyung Hee University College of Medicine, Seoul, 02447 Republic of Korea; 7 Korean Medicine Life Science, University of Science & Technology (UST), Campus of Korea Institute of Oriental Medicine, Daejeon, 34054 Republic of Korea

**Keywords:** Mild cognitive impairment, MCI, Acupuncture, Neuroimaging, Cognitive function

## Abstract

**Background:**

Mild cognitive impairment (MCI) is defined as a decline in cognitive state with preservation of activities of daily living. Medications such as donepezil and rivastigmine have been commonly prescribed for MCI, but their use is controversial. Acupuncture has been widely used in Korea and has been shown to improve cognitive function. The aim of this study is to evaluate the efficacy of acupuncture for MCI and investigate the effect of acupuncture on structural and functional brain changes in patients with MCI.

**Methods:**

This study is a randomized, assessor-blinded, sham-controlled trial. Fifty participants with MCI will be randomly assigned to the acupuncture group (*n* = 25) or sham acupuncture group (n = 25). The acupuncture group will receive acupuncture treatment at nine acupuncture points (GV20, EX-HN1, bilateral LI4, and ST36) twice a week for 12 weeks. The sham acupuncture group will receive sham acupuncture treatment at the same points with non-penetrating sham needles. Both groups will be restricted from all other treatments for the improvement of cognitive function. The primary outcome measure is the Digit Span Test (DST). The secondary outcome measures are the Digit Symbol Substitution Test (DSST), Korean version of Montreal Cognitive Assessment (MoCA-K), Seoul Neuropsychological Screening Battery-II (SNSB-II), Beck Depression Inventory-II (BDI-II), State-Trait Anxiety Inventory (STAI), working memory (WM) task performance score, and structural/functional brain changes. Outcomes will be assessed at screening, baseline, 4 and 8 weeks, and after the end of treatment. We will also observe adverse events. In the statistical analysis, a full analysis set and per-protocol analysis will be performed.

**Discussion:**

This randomized clinical trial aims to examine the efficacy of acupuncture treatment for MCI. Neuropsychological tests, psychological inventories for measuring depression and anxiety, and magnetic resonance imaging will be performed to investigate the underlying neurological mechanisms and the association between cognition, emotion, and brain networks following acupuncture treatment. The results of the trial will provide evidence supporting the efficacy of acupuncture and also add to the neurobiological understanding of acupuncture treatment for MCI.

**Trial registration:**

Clinical Research Information Service, KCT0002896. Registered on 25 May 2018.

**Electronic supplementary material:**

The online version of this article (10.1186/s13063-019-3446-9) contains supplementary material, which is available to authorized users.

## Background

Mild cognitive impairment (MCI) is an intermediate stage between normal aging and dementia. In MCI, there is an objective cognitive decline but independence in daily activities is preserved [1]. The prevalence rate of MCI varies from 3% to 42% in the elderly [2]. MCI is one of the predictive factors for Alzheimer’s disease (AD), and the annualized rates of conversion from MCI to AD range from 7.5% to 16.5% [3]. Because of its high conversion rate, both clinicians and researchers are interested in MCI as a parameter for the early detection and prevention of AD.

The diagnostic criteria for MCI have been updated over the past 20 years [4–7]. The most widely used criteria, suggested by Petersen et al. [5] include (1) objective memory impairment for the patient’s age, (2) essentially preserved general cognitive function, (3) largely intact functional activities, and (4) no dementia. In patients affected by MCI, working memory (WM) impairment and episodic memory deficits are commonly observed [8–10]. Because deficits in WM could be a predictor of progression to AD, evaluating and monitoring changes to WM in MCI is very important [11]. In addition, neuropsychiatric symptoms, such as depression and anxiety, are also predictors of dementia in MCI [12].

Despite the importance of MCI management, the therapeutic options are limited. According to the 2017 American Academy of Neurology (AAN) guidelines on MCI, there is no appropriate pharmacological option, and only regular exercise and cognitive training can be recommended for patients with MCI [13]. Even though cholinesterase inhibitors such as donepezil, rivastigmine, and galantamine are routinely recommended for AD, Lewy body dementia, and Parkinson’s disease-associated dementia [14], they are not indicated for MCI because they do not have a clinically meaningful effect and actually increase the incidence of gastrointestinal adverse events [15, 16]. As alternatives, antioxidants, such as vitamin E; nootropics, such as piracetam; and vasodilators, such as nimodipine and *Ginkgo biloba*, are used for MCI. However, the evidence to support their use is still weak [17, 18].

Acupuncture treatment is widely used in Asia and can be considered an alternative treatment for MCI. Systematic reviews have suggested that acupuncture may improve cognitive function in patients with vascular cognitive impairment, no dementia (VCIND) [19, 20], and amnestic MCI [21]. Clinical trials have shown that acupuncture treatment with electrical stimulation is more effective than nimodipine in patients with MCI [20, 22–24].

Several neuroimaging studies have examined the change in activity of brain regions or functional networks related to memory after acupuncture treatment; however, in those studies, a single acupoint was stimulated [25–28]. These acupuncture procedures are limited by the fact that they do not reflect real world practice, because acupuncture treatment is usually performed at multiple acupoints. A recent study has demonstrated that pragmatic acupuncture treatment with multiple acupoints improves cognitive function and increases the connectivity of functional networks in the brain [29].

Thus, we plan for the duration of acupuncture treatment to be longer than a previous study [29], and expect to elucidate its underlying neurological mechanism, particularly focusing on WM. In this study, we will evaluate the efficacy of acupuncture treatment compared to sham acupuncture. To help understand the mechanism by which acupuncture may improve cognition, we will assess cognitive function, emotional state, and structural and functional brain changes following acupuncture treatment.

## Methods

### Objectives

The aims of this study are (1) to assess the specific effect of acupuncture treatment on MCI in comparison with sham acupuncture and (2) to investigate the neurological mechanism of acupuncture treatment for MCI associated with improvement of cognition, based on magnetic resonance imaging (MRI) results.

### Design

**Fig. 1:**
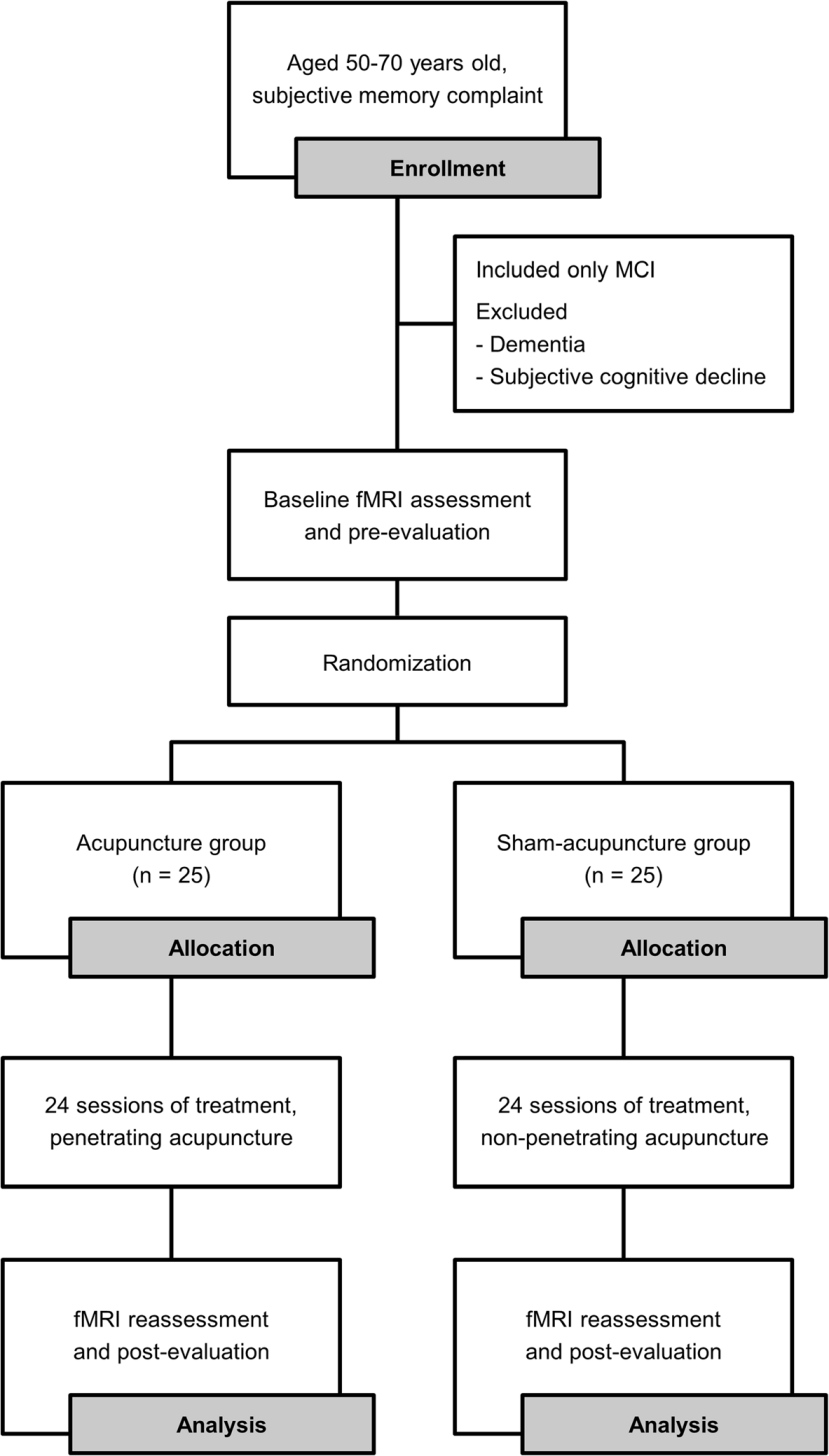
Diagram of the study flow. MCI, mild cognitive impairment; fMRI, functional magnetic resonance imaging

**Fig. 2:**
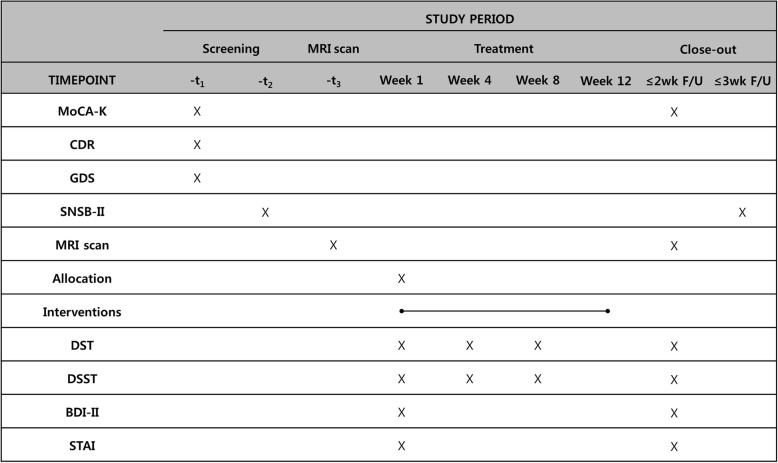
Standard Protocol Items: Recommendations for Interventional Trials Statement (SPIRIT). Overview of study process and outcome assessment. BDI-II, Beck Depression Inventory-II; CDR, Clinical Dementia Rating; d, day; DSST, Digit Symbol Substitution Test; DST, Digit Span Test; GDS, Global Deterioration Scale; MoCA-K, Korean version of Montreal Cognitive Assessment; MRI, magnetic resonance imaging; SNSB-II, Seoul Neuropsychological Screening Battery-II; STAI, State-Trait Anxiety Inventory; wk., week; F/U, follow up

This study will be a randomized, assessor-blinded, and sham-controlled trial (Fig. 1). This trial will be conducted at the Kyung Hee University Hospital at Gangdong (KHUGD) and Kyung Hee University Medical Center (KHUMC) in Seoul, Korea, beginning in June 2018. This protocol is presented in accordance with the Standard Protocol Items: Recommendations for Interventional Trials (SPIRIT) 2013 statement (see Additional file 1) and the SPIRIT figure (Fig. 2). The organizational structure and responsibilities of the researchers are shown in Additional file 2.

### Ethical considerations

The Institutional Review Boards of the Kyung Hee University Hospital at Gangdong and Kyung Hee University Medical Center approved the study (KHNMCOH 2017–09–007-001 and KOMCIRB-170915-HR-037, respectively). Ethics approval has been obtained from the Institutional Review Board (IRB) of both clinical trial institutes. This trial complies with the Declaration of Helsinki. Each hospital’s clinical trial manager will obtain informed consent after explaining the purpose and content of the study, rights of participants, and confidentiality. Demographic characteristics, vital signs, laboratory test results, past medical history, neuropsychological test results, questionnaire results, MRI results, and informed consent forms will be collected. The data will be kept as an electronic file with a password or a document in a locked cabinet. The data will be submitted to the Sponsor, the Korean Institute of Oriental Medicine (KIOM), within 1 month of the conclusion of the study, and the investigators will keep a copy. The documents will be stored for 3 years, after which they will be shredded. Any modifications of the protocol will be approved by the IRB and updated at the protocol registration site and with the journal. If the modifications result in changes in how the patients may benefit or be harmed, trial participants will be also notified of these changes.

The research team will communicate trial findings using de-identified study information via publication in a peer-reviewed journal, and assistance of professional writers is not anticipated. No later than 2 years after completion of this trial, we will deliver a completely de-identified data set to an appropriate data archive for sharing purposes.

### Participants

#### Assessment of eligibility

Eligibility for this clinical trial will be assessed in two steps. First, a Korean version of the Montreal Cognitive Assessment (MoCA-K), Clinical Dementia Rating (CDR), and Global Deterioration Scale (GDS) will be evaluated. In eligible participants, we will then assess cognitive function using the Seoul Neuropsychological Screening Battery-II (SNSB-II), on a separate day.

#### Inclusion criteria

Inclusion criteria for patients with MCI are as follows: (1) patients aged 50–70 years, (2) patients meeting the Peterson diagnostic criteria for MCI, (3) duration of memory loss longer than 3 months, (4) educational level longer than 6 years, (5) a MoCA-K score < 23, (6) a CDR score of 0.5 and GDS grade 2–3, (7) a Modified Hachinski Ischemic Score (HIS) score ≤ 4, and (8) patients who voluntarily sign the informed consent form.

#### Exclusion criteria

Exclusion criteria for patients with MCI include the following: (1) diagnosis of dementia using diagnostic criteria from the *Diagnostic and statistical manual of mental disorder, fifth edition* (DSM-5), (2) presence of cerebral lesions or brain damage, (3) history of cerebral hemorrhage or cerebral infarction, (4) neurological disorders such as Parkinson’s disease, epilepsy, brain tumor, etc., (5) mental disorders such as major depressive disorder, schizophrenia, bipolar disorder, etc., (6) subjects who have participated in other clinical trials within the previous 4 weeks, (7) subjects who have received acupuncture treatment for cognitive impairment within the previous 4 weeks, (8) subjects using medications related to dementia (as an exception, patients with a diagnosis of MCI who have been prescribed anti-dementia medications are allowed to be included in the study after a 15-day wash-out period), (9) women who are pregnant, breastfeeding, or childbearing and do not use adequate contraception, (10) presence of typical contraindications for MRI (e.g., obstructive phobia or metal implantation), (11) subjects who have experienced hypersensitivity reactions after acupuncture treatment or are unable to cooperate with acupuncture treatment, and (12) other subjects judged by the researchers as inappropriate to participate.

#### Discontinuation criteria

Discontinuation criteria for patients with MCI include the following: (1) subjects taking additional medication, traditional herbal medicine, or acupuncture for cognitive improvement during the acupuncture treatment of this trial (acupuncture and over-the-counter medicine for other purposes not related to cognitive improvement will be allowed up to twice a week), (2) subjects who do not attend the treatment for more than four consecutive times or for more than eight times in total, (3) improving or worsening of the disease, (4) surgery or hospitalization due to accident or other diseases, (5) serious adverse events, (6) participant request, and (7) others judged by the principal investigator as inappropriate to participate. To promote participant retention and adherence, we will send a text message the day before an appointment, as a reminder.

### Recruitment

A total of 50 participants diagnosed with MCI will be recruited from KHUGD and KHUMC. The participants will be recruited through advertising posters displayed in the hospitals and published in local newspapers, and banner advertisements in the subway, from June 2018.

### Randomization, allocation concealment, and blinding

Subjects will be randomly assigned to either the acupuncture or sham acupuncture group. Stratified block randomization will be used to control the influence of age and sex. An independent statistician who is not related to the intervention practice and outcome assessment will generate a random sequence using SAS® Version 9.4 (SAS Institute, Inc., Cary, NC, USA). This study will be conducted using a central randomized competitive recruitment method. Participants signing the informed consent form voluntarily and meeting the inclusion criteria will be eligible for randomization. An investigator will then assign the participants to groups based on the random sequence prepared and sent by an independent statistician via e-mail.

In this clinical trial, participants and assessors will be blinded to treatment allocation to decrease the risk of bias from the assigned acupuncture treatment group. However, practitioners will not be blinded. The participants will be informed that they will be treated either by real or sham acupuncture before they sign the informed consent form. The validity of the participants’ blinding will be assessed by an investigator who is not involved in either the patients’ allocation procedure or performance of the acupuncture treatment. The assessor will be blinded to the treatment group and prevented from speaking with the participants. Unblinding should be determined on a case-by-case basis and considered only in a critical medical emergency.

### Interventions

Acupuncture will be performed by specialists in Korean medicine following the details of the Standards for Reporting Interventions in Clinical Trials for Acupuncture (STRICTA) 2010 checklist (Table 1) [30]. Patients in both the acupuncture and sham acupuncture groups will receive 24 acupuncture treatments over 12 weeks at nine acupoints (GV20, EX-HN1, bilateral LI4, and ST36). In the sham acupuncture group, the acupuncture needle (a Streitberger needle) will not penetrate the skin. Streitberger needles are widely used as placebo needles in clinical trials [31–33]. During this trial, any other treatment for the improvement of cognitive function will be restricted in both acupuncture and sham acupuncture groups.

**Table 1:** Details of acupuncture intervention

Item	Detail	Description
Acupuncture rationale	(1a) Style of acupuncture	Korean body acupuncture
	(1b) Reasoning for treatment	We selected the optimal treatment regimen based on university textbooks about the meridian and acupuncture, and a literature review of clinical trials investigating acupuncture therapy for mild cognitive impairment and dementia
	(1c) Extent to which treatment varies	None
Details of needling	(2a) Number of needle insertions per subject per session	9
	(2b) Names of points used (unilateral/bilateral)	GV20, EX-HN1, LI4 (bilateral), ST36 (bilateral)
	(2c) Depth of insertion	5–10 mm
	(2d) Response sought	None
	(2e) Needle stimulation	Manual
	(2f) Needle retention time	15 min
	(2 g) Needle type	0.30 × 30 mm, sterilized stainless steel needle (Asia-med GmbH & Co. KG, Germany)
Treatment regimen	(3a) Number of treatment sessions	24 sessions
	(3b) Frequency and duration of treatment sessions	2 sessions/week for 12 weeks
Other components of treatment	(4a) Details of other interventions administered to the acupuncture group	None
	(4b) Setting and context of treatment	Hospital outpatient department
Practitioner background	(5) Description of participating acupuncturists	Specialist in oriental neuropsychiatry or longer than 1-year career resident under the guidance of an oriental psychiatric specialist
Control or comparator interventions	(6a) Rationale for the control or comparator in the context of the research question	As placebo control, non-penetrating sham acupuncture needles will be used
	(6b) Precise description of the control or comparator	Non-penetrating acupuncture treatment at the same acupoints using a Streitberger device

### MRI scanning procedure

**Fig. 3:**
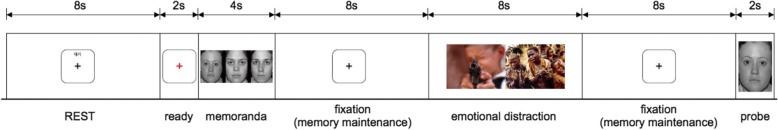
Paradigm of working memory task for a task functional magnetic resonance imaging (fMRI) run

In order to evaluate neural correlates involved in improving cognitive functions following acupuncture treatment, brain imaging data will be acquired at baseline and over a 12-week period following acupuncture treatment. Imaging data will be acquired using a 3.0-T Philips Ingenia MRI scanner (Phillips Medical System, The Netherlands) at KHUGD and a 3.0-T Philips Achieva MRI scanner (Phillips Medical System) at KHUMC, both of which are equipped with a 32-channel head coil. We will use the same imaging parameters for both MRI scanners at the two sites. First, structural MRI will be recorded for standard space co-registration, and T1-weighted 3D turbo field echo sequences (repetition time (TR)/echo time (TE) = 9.9/4.6 ms, flip angle = 8°, voxel size = 1 mm isotropic) will be applied. A whole-brain T2*-weighted gradient echo blood oxygenation level-dependent (BOLD) pulse sequence (TR/TE = 2000/35 ms, flip angle = 90°, 34 axial slices, voxel size = 1.72 × 1.72 × 4.2 mm) will be used to obtain functional MRI (fMRI) data. We will use a task fMRI approach to investigate changes in brain responses to a cognitive task following acupuncture treatment. An adaptation of the WM task by Iordan et al. [34] will be used with emotional distractors. The emotional distractors are pictures depicting emotional scenes with high arousal and negative valence from the International Affective Picture System (IAPS; mean arousal and valence scales are 6.4 and 1.8, respectively) (Fig. 3). The task fMRI run will last for 8 min and 30 s for 12 tasks. The tasks will be presented visually using MRI-compatible video goggles (NordicNeuroLab, Norway). Participants will use a push-button to perform a WM task, and WM performance will be measured by the accuracy rate and response time. Two 8-min resting-state fMRI runs will also be acquired before and after the task fMRI run. Finally, diffusion-weighted images will be obtained using a spin-echo pulse sequence (TR/TE = 9125/97 ms, voxel size = 0.96 × 0.96 × 2.2 mm, 68 slices, b-value = 1000 s/mm^2^, 32 non-collinear directions).

### Outcome measurement

#### Primary outcome

The primary outcome is change in the Digit Span Test (DST) score from baseline to the end of treatment. The DST is used to measure WM, attention, and concentration, and is included in the Wechsler Intelligence Scale [35, 36]. Participants will hear sets of numbers and be asked to recall the numbers forwards or backwards. The digit sequences will be progressively longer until participants cannot recall the sets of numbers twice for a particular sequence length. The DST score is calculated by summation of the maximum number of digits successfully recalled forwards and backwards. The DST will be conducted at baseline, 4 weeks, 8 weeks, and within 2 weeks of the end of treatment.

#### Secondary outcomes

The Digit Symbol Substitution Test (DSST) is used to measure attention and psychomotor speed, and is sensitive to cognitive changes at high levels of cognition [37]. Digit–symbol pairs (e.g., 1/−, 2/┴ ... 7/Λ, 8/X, 9/=) and a list of digits will be presented to the participants. During a 90-s period, the participants draw as many paired symbols as possible under each corresponding digit. The DSST score is the number of correct symbols within a given time. The DSST will be assessed at baseline, 4 weeks, 8 weeks, and within 2 weeks of the end of treatment.

The MoCA is a widely used screening tool for MCI. Its cutoff score was 26 in the original version [38], but currently a cutoff score of 23 is suggested for better diagnostic accuracy [39, 40]. It consists of the trail-making test-B, three-dimensional cube copy, clock drawing test (CDT), confrontation naming task, short-term memory recall task, DST, serial 7 s, repetition of sentences, semantic verbal fluency task, and verbal abstraction task. In addition, orientation to time and place is also evaluated. The Korean version of the MoCA has been validated in the clinical setting and a cutoff score of 23 has been adopted [41]. The MoCA-K will be assessed at screening and within 2 weeks of the end of treatment.

The SNSB-II is a standardized neuropsychological test battery that has been validated in Korea [42–44]. This test is useful in distinguishing MCI from subjective memory impairment or dementia. It contains various cognitive assessment tools and dementia screening and evaluation tests, such as the Korean Mini Mental State Examination (K-MMSE), Short Version of the Geriatric Depression Scale (SGDS), Korean Barthel Activities of Daily Living (K-ADL), Korean Instrumental Activities of Daily Living (K-IADL), CDR, and GDS. The SNSB-II assesses five cognitive domains: attention, language and related functions, visuospatial function, memory, and frontal/executive functions. The attention domain includes the DST. The domain of language and related functions includes partial items of the Paradise Korean Western Aphasia Battery (K-WAB), Korean Boston Naming Test (K-BNT), calculation, and praxis test. The visuospatial function domain includes drawing and Rey Complex Figure Test (RCFT) copy. The memory domain includes the RCFT immediate and delayed recalls and recognition test and Seoul Verbal Learning Test (SVLT). The frontal/executive function domain includes the Korean Color Word Stroop Test (K-CWST), contrasting program and Go-No-Go test, Fist-Edge-Palm and alternating hand movement test, and Luria loop test. The SNSB-II will be assessed at screening and within 3 weeks of the end of treatment.

The Beck Depression Inventory-II (BDI-II) is a self-reported questionnaire to evaluate the severity of depression [45–47]. It consists of 21 symptoms related to the diagnostic criteria of major depressive disorder (MDD) in DSM-IV. Each symptom is graded on a 4-point Likert scale (0–3) and the total score ranges from 0 to 63. Higher scores mean higher levels of depression. The Korean version has been validated in healthy university students [48] and patients with depression [49, 50]. The BDI-II will be assessed at baseline and within 2 weeks of the end of treatment.

The State-Trait Anxiety Inventory (STAI) is also a self-reported scale to measure two types of anxiety: state anxiety and trait anxiety [51]. It consists of 40 questions, with each question rated on a 4-point Likert scale (1–4). The state and trait anxiety scores range each from 20 to 80. Higher scores mean higher levels of anxiety. The Korean version of Form Y of the STAI has been validated in high school and university students [52]. The STAI will be assessed at baseline and within 2 weeks of the end of treatment.

### Sample size calculation

To assess the efficacy of the intervention, the minimal clinically important difference (MCID) of DST was considered. However, there was no well-established prior study to derive the MCID of DST. Thus, the effect size was calculated from a similar study examining acupuncture treatment for MCI.

The sample size is estimated based on the result from a previous study [29]. The mean difference in the DST score is assumed to be 0.87 and the standard deviation (SD) of that is assumed to be 0.89 between the acupuncture group and sham acupuncture group. Sample size calculation shows that a total of 44 patients is needed for 90% statistical power and 5% significance level. With an estimated dropout ratio of 10%, the necessary sample size is 50 patients (25 patients for each acupuncture group).

### MRI data analysis

MRI data will be processed using multiple software packages including FMRIB Software Library (FSL; http://fsl.fmrib.ox.ac.uk/fsl), Analysis of Functional NeuroImages (AFNI; https://afni.nimh.nih.gov/afni), FreeSurfer (http://freesurfer.net), and Statistical Parametric Mapping (SPM; http://www.fil.ion.ucl.ac.uk/spm).

#### Voxel-based morphometry (VBM) analysis

The structural T1-weighted MRI data will be used in a VBM analysis using SPM. Tissue classification, registration, and bias correction will be processed in a generative model. Nonlinear deformation for warping gray matter and white matter images will be determined using the Diffeomorphic Anatomical Registration using Exponentiated Lie algebra (DARTEL) toolbox [53]. Gray matter and white matter images will be smoothed and registered to the standard Montreal Neurological Institute (MNI) space.

#### Brain responses to the WM task and connectivity analyses

T2*-weighted BOLD images will be pre-processed including physiological artifact correction (AFNI, RETROICOR), head motion correction (FSL, mcflirt), and non-brain extraction (FSL, bet). Co-registration between functional and structural images will be performed using boundary-based registration (FreeSurfer, bbregister). The aligned BOLD data will then be registered to MNI space (FSL, fnirt). Spatial smoothing and temporal high-pass filtering will also be performed. General linear modeling (FSL, feat) will yield a brain response map for the WM task in each participant. For functional connectivity analyses, we will use dual-regression independent component analysis and seed-voxel correlation approaches.

#### Diffusion tensor imaging (DTI) analysis

Diffusion-weighted images will be aligned to the b_0_ image to correct for eddy current distortion and then non-brain extraction will be performed. Using FSL’s Diffusion Toolbox (FDT, FSL), a fractional anisotropy map will be computed along with the mean, radial, and axial diffusivity maps, which fit a DTI tensor model. These DTI metric maps will be aligned to the MNI space FMRIB58 FA map.

### Statistical analysis

In this study, statistical analyses will be performed by an independent statistician using full analysis set (FAS) and per-protocol (PP) analyses. The FAS refers to a set that is almost identical to the intention-to-treat (ITT) ideal of including all randomized subjects, but may exclude subjects who never received the treatment or missed baseline measurement after randomization. In addition, we will perform supplementary analysis of the PP analysis group. The PP analysis group refers to a set of subjects who completed the treatment originally allocated. Compliance in this study is deemed to be satisfied only by those who have undergone more than 70% of the total number of acupuncture treatments. In other words, out of the 24 treatments, participants who do not receive treatment more than 17 times are excluded from the PP analysis group because of violation of the protocol.

All demographic and baseline characteristics of participants will be presented based on descriptive analyses. Continuous data will be presented as the average and SD and analyzed using the independent two-sample *t* test or Wilcoxon rank sum test. Dichotomous data will be presented as frequency and percentile and analyzed using the chi-square test or Fisher’s exact test.

Post-test scores will be analyzed using analysis of covariance (ANCOVA), using each group as the fixed factor and baseline score with strata factors (such as sex and age) as the covariate. Within a group, the difference pre-treatment and post-treatment will be compared using the paired *t* test or Wilcoxon singed rank test for continuous data and the chi-square test or Fisher’s exact test for dichotomous data. For repeated measures outcomes, repeated measures analysis of variance (ANOVA) will be performed to compare differences between the times of measurement.

Statistical analyses of clinical outcomes will be performed using SAS® Version 9.4 (SAS Institute, Inc.). All tests will be two-sided and a *p* value <0.05 will be considered statistically significant. We will use multiple imputations for missing data.

Statistical comparisons of data on MRI outcomes will be performed differently. Voxel-wise generalized linear model (GLM) analysis will be performed to compare gray matter volumes within and between groups while controlling for age and sex. Statistical maps of comparisons in gray matter volumes will be corrected for multiple comparisons using the false discovery rate (FDR, *p* < 0.05). Similar to gray matter volumes, differences in brain responses to the WM task and functional connectivity from the resting state fMRI at baseline will be assessed between groups using the independent samples *t* test and voxel-wise GLM analysis (FSL, feat). fMRI metrics between baseline and post-treatment will be compared using the paired samples *t* test in a voxel-wise GLM. Age and sex will be added to the GLM as regressors of no interest. Correction for multiple comparisons of fMRI metrics will be performed using a corrected cluster-size threshold of *p* < 0.05. Statistical comparisons The DTI metric maps will be statistically compared within and between groups using a non-parametric permutation test with 5000 permutations. For multiple comparisons across voxels in DTI metric maps, family-wise error correction will be used with threshold-free cluster enhancement (Randomise, FSL). Whole-brain linear regression analyses will be performed to test association between metrics of cognitive function and changes in brain function and structure. This trial will enroll 50 patients with MCI and this could be a cohort with heterogeneous cognitive function. Thus, we will follow up the aforementioned analyses to evaluate brain function and structure in subgroups with different cognitive functions at baseline. We will divide patients with MCI into equal sample sizes of low-cognitive and high-cognitive function subgroups using the MoCA-K. We will compare between MRI outcomes for low-cognitive and high-cognitive function to test whether cognitive function affects brain function and structure groups, using the independent samples *t* test with identical multiple comparisons correction, as previously described.

### Safety

Adverse events (AEs) will be recorded at each visit during the trial. Among the AEs, the following cases are defined as serious AEs (SAEs): (1) death or threat to life, (2) hospitalization (initial or prolonged), (3) disability or permanent damage, (4) congenital anomaly/birth defect, and (5) other important medical events. If SAEs occur, the principal investigator should notify the Sponsor within 24 h. If a participant reports an AE or SAE, the investigators evaluate whether the AEs or SAEs are related to the intervention. If required, appropriate medical management should be provided under the usual victims’ compensation principles. If the adverse event is too serious to continue the trial, the participant will be withdrawn. However, if the AE or SAE is probably not or definitely not related to the intervention and the harm is not serious, the participant will not be withdrawn.

The AEs and SAEs will be recorded for safety assessment. Given the safety of acupuncture, we did not pre-define a safety endpoint, as it was deemed unlikely that a large number of meaningful AEs would be observed.

### Data management

In this trial, all data will be recorded electronically. This may be done at the participating site where the data originated. A web-based system for collecting trial data will have programs designed to detect missing data or specific errors. If these errors are detected, a warning sign will be displayed next to coding boxes and a query will be generated. The investigator who receives the inquiry will correct the data after checking the original documents. Interim analyses will not be undertaken at any time during the whole period of study.

All principal investigators and research coordinators will have access to the cleaned final data sets. Project data sets will be housed at https://kiom1.mdsol.com/MedidataRave, and all data sets will be password protected.

### Monitoring

This study is an investigator-initiated trial, which explores the acupuncture mechanism and efficacy. A data monitoring committee (DMC) was not considered necessary in terms of safety concerns, outstanding benefits, and futility, as is often the case in similar studies.

The monitors, who are working in other independent divisions of the KIOM organization, will monitor source documents at all sites and will conduct at least one onsite-monitoring visit every 2 months. The monitors will review the source documents as needed, to determine whether the data reported in the Web-based system are complete and accurate. Source documents are defined as medical charts, associated reports, and records. The monitors will confirm that the regulatory binder is complete and that all associated documents are up to date. The regulatory binder should include the protocol and all revisions of informed consent, IRB approvals for all of the above documents, IRB correspondence, case report forms, and the investigator’s agreements.

## Discussion

To prevent or slow the progression of dementia, it is important to manage it in its early stage (i.e., the MCI state). Unfortunately, there are no recommended medications for MCI because cholinesterase inhibitors, well-known anti-dementia drugs, have more AEs than benefits when prescribed to patients with MCI [13–16]. In this situation, acupuncture therapy can help improve the cognitive function of patients with cognitive decline [19–24]. However, studies investigating the effects of acupuncture treatment in patients with MCI have compared acupuncture to conventional anti-dementia drugs (not sham acupuncture), and usually tested single acupoint stimulation, which is not commonly used in practice to treat patients. To assess the efficacy of acupuncture in MCI, we designed a randomized, assessor-blinded, sham-controlled trial. An acupuncture treatment protocol was developed through the consensus of investigators, to reflect actual practice.

Studies have identified abnormalities in the structure and function of the brain in patients with MCI compared to age-matched healthy adults [26, 54]. The association between progression of cognitive decline and MRI metrics including Aβ and Tau in the cerebrospinal fluid and gray matter volumes in the regions of the brain is known to play a role in cognitive function [55].

In this trial, we aim to investigate differences in functional brain responses to the WM task, functional brain networks, and brain structures following acupuncture treatment in patients with MCI. In order to clarify the efficacy of acupuncture, we will compare changes in MRI metrics between the acupuncture group and sham acupuncture group, and test whether improved cognitive function is associated with changes in MRI metrics following acupuncture treatment [54]. Although a few studies have investigated structural and functional brain plasticity in patients with MCI following acupuncture stimulation [25, 26, 56], to our knowledge, our study will be the first randomized sham-controlled trial reflecting actual practice (multiple acupoints and 24 treatment sessions) using MRI.

As described previously, we will especially focus on working memory, among different cognitive functions. Working memory deficits are already present in patients with MCI [57–59], and it is a predictor of negative prognosis [58]. In addition, working memory training can improve global cognitive functions in early AD [60]. Acupuncture can also affect working memory [61, 62]; thus, we expect that acupuncture treatment will improve cognitive function in MCI through improvement of working memory. Because our aim is to show the neural mechanism and the efficacy of acupuncture treatment, DST, not a measure of global cognitive function, has been selected as a primary outcome measurement. DST is commonly used to evaluate working memory [63]. A DST score will be obtained every 4 weeks to detect changes.

Cognitive decline in the elderly is also closely related to emotional problems such as depression and anxiety. In the depressive or anxious state, cognitive function declines and psychomotor retardation or agitation is observed. In MCI, high levels of depression and anxiety have an influence on the progression from MCI to dementia [12]. Thus, we will examine not only cognitive function but also the emotional state in participants with MCI. In addition, we will have a WM task fMRI run with emotional distractors and will then examine the brain responses to high arousal and negative valence pictures and the correlation between emotionally evoked brain responses and patients’ emotional rating scores.

The limitations of the study should be noted. We adopted Streitberger sham acupuncture as a control. The Streitberger sham needle has been designed for reliable control treatment in acupuncture research [64]. However, not only the Streitberger needle, but any type of sham acupuncture has limitations. First, we cannot blind the practitioner to the treatment allocation (our trial is not a double-blinded trial). The practitioner should be aware of the treatment type, and then they will push the shaft of needle into the handle for sham acupuncture as they were trained beforehand. In order to minimize the risk of bias as much as possible, we will carefully blind the participants and assessors to acupuncture type. Second, sham acupuncture often shows large non-specific effects [64–66] and produces physiological activity [67]. A non-specific effect of sham acupuncture cannot be dismissed in this trial, but we may compare the differences in neurological mechanisms between the two groups. Thus, we may identify the acupuncture-specific pathway associated with the improvement of cognition based on the MRI results even if the total (specific plus placebo) effect is not significantly different. Third, even if we wanted to consider MCID as a primary outcome, only MCIDs in the MMSE [68, 69] and Alzheimer’s Disease Assessment Scale-Cognitive Subscale (ADAS-Cog) [70, 71] for dementia have been studied. In those studies, the duration of interventions for neurocognitive disorders was much longer than in this trial. Thus, the MCID in the DST score for MCI cannot be included in this design. For establishing the MCID, it the acupuncture needs to be used for a longer period and interviews with participants or the Delphi expert consensus method are applied. Therefore, we calculated the effect size based on a previous study [29]. To our knowledge, there are several RCTs examining acupuncture for treatment of MCI [21], but Tan et al. [29] compared acupuncture to sham control and used the DST as an outcome measurement. Although the number of participants is small, it would be an appropriate reference to our study when considering clinical relevance. Finally, we have no healthy group as a control in this trial. A previous meta-analysis [72] showed differences between brain networks in MCI and healthy participants; however, our trial focuses on investigating acupuncture-induced functional and structural brain changes, not MCI-specific characteristics of brain function/structure relative to healthy controls. Thus, we will compare brain network changes between the acupuncture and sham acupuncture groups.

## Trial status

The study was registered with the Clinical Research Information Service on 25 May 2018, registration number KCT0002896. This protocol is based on version 3.2 of 6 March 2019. Recruitment of participants started on 8 June 2018 and the first participant was enrolled on 18 July 2018. Recruitment will continue until November 2019. The items from the World Health Organization Trial Registration Data set are summarized in Table 2.

**Table 2:** The items from the World Health Organization Trial Registration Data Set

Data category	Information
Primary registry and trial identifying number	CRIS KCT0002896
Date of registration in primary registry	25 May 2018
Sponsor	KIOM
Contact for public/scientific queries	JHL, KMD, Ph.D (omdjun@kiom.re.kr)
Public title	None
Scientific title	Neurocircuitry of Acupuncture Effect on Cognitive Improvement in Patients with Mild Cognitive Impairment Using Magnetic Resonance Imaging: A Study Protocol for a Randomized Controlled Trial
Countries of recruitment	Republic of Korea
Health condition(s) or problem(s) studied	MCI
Intervention(s)	Active comparator: acupuncture
	Sham comparator: non-penetrating sham acupuncture
Key inclusion and exclusion criteria	Ages eligible for study: 50–70 years; sexes eligible for study: both; accepts healthy volunteers: no
	Inclusion criteria: diagnosis of MCI, MoCA-K < 23, CDR score of 0.5, GDS grade 2–3
	Exclusion criteria: diagnosis of dementia, neurological disorders, mental disorders
Study type	Interventional
	Allocation: randomized; intervention model: parallel assignment; blinding: subject-assessor blinding
	Primary purpose: treatment
Date of first enrolment	18 July 2018
Target sample size	50
Recruitment status	Recruiting
Primary outcome(s)	DST
Key secondary outcomes	DSST, MoCA-K

*CRIS* Clinical Research Information Service, *DSST* Digit Symbol Substitution Test, *DST* Digit Span Test, *MCI* mild cognitive impairment, *MoCA-K* Korean version of Montreal Cognitive Assessment, *KIOM* Korean Institute of Oriental Medicine

## Additional files

Additional file 1: SPIRIT 2013 Checklist: Recommended items to address in a clinical trial protocol and related documents. (DOCX 61 kb)

Additional file 2: Organizational structure and responsibilities. (DOCX 17 kb)

## Data Availability

Not applicable.

## Abbreviations

AAN: American Academy of Neurology
AD: Alzheimer’s disease
ADAS-Cog: Alzheimer’s Disease Assessment Scale-Cognitive Subscale
AE: Adverse events
AFNI: Analysis of functional neuroimages
ANCOVA: Analysis of covariance
ANOVA: Analysis of variance
BDI-II: Beck Depression Inventory-II
BOLD: Blood oxygenation level-dependent
CDR: Clinical dementia rating
CDT: Clock drawing test
DARTEL: Diffeomorphic anatomical registration using exponentiated lie algebra
DSM-5: Diagnostic and statistical manual of mental disorder, fifth edition
DSST: Digit Symbol Substitution Test
DST: Digit Span Test
DTI: Diffusion tensor imaging
FAS: Full analysis set
FDR: False discovery rate
FDT: FMRIB software library’s diffusion toolbox
fMRI: Functional magnetic resonance imaging
FSL: FMRIB software library
GDS: Global Deterioration Scale
GLM: Generalized linear model
HIS: Hachinski Ischemic Score
IAPS: International Affective Picture System
IRB: Institutional Review Board
ITT: Intention-to-treat
K-CWST: Korean Color Word Stroop Test
K-ADL: Korean Barthel Activities of Daily Living
K-BNT: Korean Boston Naming Test
KHUGD: Kyung Hee University Hospital at Gangdong
KHUMC: Kyung Hee University Medical Center
K-IADL: Korean Instrumental Activities of Daily Living
KIOM: Korean Institute of Oriental Medicine
K-MMSE: Korean Mini Mental State Examination
K-WAB: Paradise Korean Western Aphasia Battery
MCI: Mild cognitive impairment
MCID: Minimal clinically important difference
MDD: Major depressive disorder
MNI: Montreal Neurological Institute
MoCA-K: Korean version of Montreal Cognitive Assessment
MRI: Magnetic resonance imaging
PP: Per-protocol
RCFT: Rey complex figure test
SAE: Serious adverse event
SD: Standard deviation
SGDS: Short Version of the Geriatric Depression Scale
SNSB-II: Seoul Neuropsychological Screening Battery-II
SPM: Statistical parametric mapping
STAI: State-Trait Anxiety Inventory
STRICTA: Standards for Reporting Interventions in Clinical Trials for Acupuncture
SVLT: Seoul Verbal Learning Test
TE: Echo time
TR: Repetition time
VBM: Voxel-based morphometry
VCIND: Vascular cognitive impairment, no dementia
WM: Working memory
