# Supplementary material for: Neurocircuitry of acupuncture effect on cognitive improvement in patients with mild cognitive impairment using magnetic resonance imaging: a study protocol for a randomized controlled trial
Source: Trials. 2019 May 30;20:310. doi: 10.1186/s13063-019-3446-9 (PMC6543690; doi:10.1186/s13063-019-3446-9)
Supplement: Supplementary file 2 — Organizational structure and responsibilities. (DOCX 17 kb) [file 13063_2019_3446_MOESM2_ESM.docx]

**Organizational structure and responsibilities**

| Principal Investigator and  Research Physician | Trial Management Committee (TMC)  (Principle investigator, Research Physician, Administrator) | Data Manager |
| --- | --- | --- |
| Design and conduct of trial  Preparation of protocol and revisions  Preparation of eCRFs (Case Report Forms)  Organizing trial management committee meetings  Managing CTO [Clinical Trials Office]  Publication of study reports  Members of TMC [Trial Management Committee]  Agreement of final protocol | Study planning  Reviewing progress of study and if necessary agreeing changes to the protocol  Provide annual risk report to ethics committee  SUSAR [Serious unexpected suspected adverse events] reporting  Responsible for trial master file  Budget administration and contractual issues  Data verification  Randomization  Organization of neuroimaging data collection | Maintenance of trial IT system and data entry  Data verification |
